# How Problems with Microplastics in Research and Application Can be Overcome: Lessons From the Experience of Plastics Stakeholders

**DOI:** 10.1002/gch2.202500271

**Published:** 2025-09-27

**Authors:** Damjana Drobne, Gabriela Kalčikova, Ulrike Braun, Marcus Lukas, Rudolf Reuther, Lesley Tobin, Bernd Giese

**Affiliations:** ^1^ Biotechnical Faculty University of Ljubljana Ljubljana 1000 Slovenia; ^2^ Faculty of Chemistry and Chemical Technology University of Ljubljana Ljubljana 1000 Slovenia; ^3^ Faculty of Mechanical Engineering Brno University of Technology Brno 61669 Czech Republic; ^4^ Federal Environment Agency 12307 Berlin Germany; ^5^ ENAS Environmental Assessments 77886 Lauf Germany; ^6^ Optimat Glasgow G2 1PP UK; ^7^ Institute of Safety and Risk Sciences (ISR) BOKU University Vienna 1190 Austria

**Keywords:** additives, design, harmonization, microplastics, nanoplastics, risk assessment, standardization

## Abstract

The potential human health and environmental risks of micro‐ and nanoplastics (MNPs) have received increasing scientific attention in recent years. However, methodological challenges, particularly in hazard identification and exposure assessment, continue to hinder reliable risk characterization of MNPs. Taking into account the often complex composition of MNPs as a mixture of polymer, additives, and possible chemical and biological contaminants, comprehensive and robust assessments based on reproducible and harmonized protocols are needed before evidence‐based regulatory decisions can be made. Furthermore, given the increasing amounts of plastics in various applications, it is already necessary to develop risk‐mitigating strategies aimed at minimizing potential adverse effects on ecosystems and human health. To address 1) the need for better networking of research and standardization across the entire analytical workflow and 2) the requirements for risk‐minimizing design and labeling of plastics, an expert workshop was held in the course of a Horizon 2020 research and innovation project, PlasticsFatE. The results of the event contain concrete recommendations, such as 1) a graduated approach to methodological unification with harmonization in particle and hazard analysis and standardization in monitoring, 2) a risk‐minimizing design of plastics, and 3) the obligation to label toxic substances.

## Graphical abstract

This perspective addresses the methodological challenges that continue to hinder reliable risk characterization of micro‐ and nanoplastics. It also discusses risk‐mitigating strategies aimed at minimizing potential adverse effects on ecosystems and human health. Specific recommendations are given, such as 1) an approach to methodological harmonization, 2) a risk‐minimizing design of plastics, and 3) the obligation to label toxic substances.

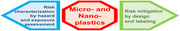

## 1 Introduction

Microplastic particles in environmental media and the risk of contamination of food and drinking water are a critical consequence of our massive use of plastic products and the associated release of this universally applicable material. The term “microplastics” is not generally harmonized and–depending on the definition source^[^ ^1^, ^2^, ^3^, ^4^ ^]^–includes particles whose longest dimension ranges from 100 nm to 5 mm. There are separate definitions for plastic fibers, which also take the aspect ratio into account and therefore also include fibers up to a length of 15 mm. Consequently, the term microplastics includes a nanofraction whose physico‐chemical properties are expected to differ from the micrometer‐sized plastic particles.^[^ ^5^ ^]^ In the following, we will thus speak of micro‐ and nanoplastics (MNPs). An increasing number of publications on the environmental and health impacts of MNPs have been published in recent years.^[^ ^5^, ^6^, ^7^, ^8^, ^9^, ^10^, ^11^ ^]^ The analysis of environmental and biota samples gives the impression that MNPs are distributed globally by now.^[^ ^12^, ^13^, ^14^ ^]^ And there is increasing evidence that they can also enter human tissue.^[^ ^11^, ^15^ ^]^ While exposure to MNPs is alarming, knowledge about their hazard potential for humans is still fragmentary.^[^ ^8^, ^11^, ^16^ ^]^ In addition to the problem that MNPs pose a challenge for toxicology due to their complex mixture of a specific polymer, additives and a possible variety of non‐intentionally added substances,^[^ ^17^ ^]^ the assessment of the risk to humans is also complicated by lack of appropriate protocols for hazard analysis, in the form of a characterization of the particles and their toxicological profile, and too little harmonization of monitoring approaches for MNPs in environmental media, food and beverages and biota samples.^[^ ^14^, ^18^ ^]^ The latter limits the comparability and thus the informative value of the results. Above all, to ensure that the toxicological analysis is based on the actual hazard conditions, we need to know what kind of particles we are exposed to in environmental media, food, and beverages. Analytics and toxicology are the two areas of MNPs research where progress could bring more clarity about the risks of MNPs, which are likely to be present for a very long time and possibly even increase, despite efforts to reduce plastic emissions.^[^ ^7^, ^19^ ^]^ The PlasticsFatE (Plastics Fate and Effects on human health) research project aims to improve our current knowledge of the effects of MNPs and their associated additives or adsorbed contaminants (A/C) in the human body. Together with four other projects, PlasticsFatE is funded by the European Commission to support the implementation of the European Union (EU) Strategy for Plastics in a Circular Economy.^[^ ^20^ ^]^ Work in these projects has shown that for the analysis of microplastics in complex matrices, harmonized sampling, sample processing, and detection methods do not exist. For example, there are currently only international standards with very general information^[^ ^1^, ^2^ ^]^ or very specific applications, e.g., for washing processes.^[^ ^21^ ^]^ For smaller particles in the nanometer range, only very sophisticated and complex detection methods exist, which are often time‐consuming and expensive. This is due to the fact that the most commonly used instruments for determining plastic particles are limited by physical‐technical requirements, such as spectroscopic microscopy for particles in the nano size range, or by detection limits, such as in thermo‐analytical methods for mass loads below the µg range in the sample, which are difficult to achieve for nanoparticles due to their low mass. Alternative detection methods for particles below 1 µm are electron microscopy, (field) flow detectors, or dynamic light scattering, all of which, however, cannot distinguish between the particle composition and therefore require extensive sample preparation. There are also several challenges when testing the toxicity of MNPs in particularly with regard to the choice of reference materials, the analytics of MNPs during testing, as well as the selection of biological tests and endpoints to be analyzed. Important to know is also, how realistic the current toxicological tests are to predict the hazard potential of MNPs for humans. For this reason, analytics and toxicology were selected as topics for a workshop with experts representing different stakeholders related to development and production, application, and risk assessment of plastics to collect suggestions for necessary measures to improve the risk assessment of MNPs. The workshop, entitled “Stakeholders’ input in relation to standardization and regulation of microplastics” was organized by PlasticsFatE partners on November 29th, 2023, in Frankfurt, Germany. The final purpose of this meeting was to inform key policymakers, industry, and standardization bodies about essential stakeholder requirements for risk assessment and management of plastics to finally shape future EU policy.

## 2 Topics and Stakeholder Input

To achieve the objectives of the workshop, the discussions were primarily focused on knowledge gaps and barriers in terms of 1) analytics and 2) toxicology related to MNPs to discuss which problems hinder risk assessment of MNPs and which measures and methods can improve it.

However, potentially hazardous effects, and the lack of knowledge about the effects of certain plastics in their contexts of use can already be reduced on the one hand by appropriate selection and design of the polymer material, including its additives, and on the other hand by labeling its ingredients. Plastics are currently labelled only by specifying the main polymer from which the plastic is made. But plastics can contain a number of additives, and many of the additives used are classified as hazardous to humans and the environment. With regard to the selection and design of plastics, it would be helpful to know whether it is possible to establish indicators for the prediction of the hazard potential of MNPs that would help in decision‐making at early stages of product design or approval. For these reasons and also because the mass of plastic use is increasing,^[^ ^22^, ^23^ ^]^ this event included a third topic, namely, what can already be done to reduce the potential risk of MNPs to humans through upstream measures 3). This topic addressed lessons learned for a risk‐minimizing design as well as regulatory needs for risk‐oriented labeling of plastics.

A World Café format supports the in‐depth discussion of topics by subsequent rounds of discussion with changing participants.^[^ ^24^ ^]^ It was chosen for the workshop because it facilitates dialogue and helps to collect the views of participants in a short period of time.^[^ ^25^ ^]^ With its subsequent discussions, it maximizes the opportunity to draw on different stakeholder perspectives in differently composed groups that help to avoid uniform conversation settings.

Based on a list of contacts provided by the partners of the project PlasticsFatE, participants of the workshop were invited to reflect a broad variety of societal actors involved in the development, production, application, and risk assessment of plastics. In addition, expertise in the standardization of methods and materials necessary for risk assessment should be represented at the workshop by at least one of the participants. The number of participants (including the organizers) was capped at 21. Of the 21 participants in the workshop (incl. the organizers), five were from academia, five from industry or industry associations, five from government agencies involved in the analysis of MNPs and their toxicology, two from companies providing analytical services for plastics, one from a private institute specializing in standardization, and three from companies specializing in either risk assessment and monitoring of chemicals, innovation and strategy consulting, or EU research policy (**Table** **1**).

**Table 1:** List of entities represented by the workshop participants.

Academia	University of Brescia (Italy) Montanuniversität Leoben (Austria)
Industry or industry associations	BASF SE (Germany) Covestro Deutschland AG (Germany) PlasticsEurope Flexible Packaging Europe Verband der Chemischen Industrie e.V. (Germany)
Companies providing analytical services for plastics	Eurofins Analytical Services Hungary Ltd. BioDetection Systems bv. (Netherlands)
Government agencies involved in the analysis of MNPs	Max Rubner Institute – Federal Research Institute of Nutrition and Food (Germany) Norwegian Institute for Water Research (NIVA) Bundesanstalt für Materialforschung und ‐prüfung (BAM) (Germany)
Standardization	German Institute for Standardization (DIN e.V.)

## 3 Key Results

In the following sub‐sections, the most important points and outcomes of the workshop are explained. According to the three discussion topics a) MNPs analysis, MNPs toxicology, and c) design and labeling of plastics to reduce potential risks caused by MNPs, the focus of each sub‐section is on important findings as well as methodological and technical issues that have emerged with the increasing research on MNPs. Major aspects are

1. the comparability of methodologies for quantification, identification, and risk assessment of MNPs,
2. the complexity of MNPs as polymer particles with additives and possible contaminants,
3. deficits in the documentation and availability of protocols and data, and
4. the identification of critical aspects that may be relevant for the design and labeling of plastics.

### 3.1 Problems and Requirements of MNPs Analytics

Validated methods are needed to create a reliable knowledge base on the occurrence and type of MNPs. Such method validation can initially be achieved through voluntary, coordinated standard operating procedures (SOPs) established, e.g., by different laboratories or a group of interest (harmonization process). The implementation of a standardization process at, e.g., an international level goes further and is subject to more stringent, generally accepted procedures and guidelines between different countries and stakeholders (so‐called experts).

Standards, as an outcome of standardization, are the basis for regulation/monitoring programs or for limit values and are generally accepted by politicians and authorities. However, creating a new standard is neither an easy task nor is it quickly achievable. The development of a standard usually takes at least 3 years, which means that standards are mostly lagging behind current research. This long time span is because standardization is a very formalized process requiring a consensus between the experts. Establishing preceding harmonization protocols would accelerate the “consensus‐dominated” standardization process, therefore.

Politics often demands fast solutions, especially when it is needed for regulatory purposes. Thus, on the level of technology governance—in other words, policymaking and safety guidance—an initiative to push forward the necessary standards in MNPs analytics is urgent. What is more, MNPs are needed to be monitored in different environmental matrices such as soil, water, and air as well as in biological samples, e.g., blood and urine, or in food.

The challenges of complex matrices make it necessary to even have a number of standards that may also need to be adapted to the ranges of the respective microplastic particles. Ultimately, a separate standard is needed for each matrix, addressing sampling and sample preparation dependent on different detection tools (that is, particle numbers, mass fraction). First examples for harmonization and standardization are the standardized methods for extraction and analysis of microplastics in drinking water developed by the Californian State Water Board, in collaboration with the Southern California Coastal Water Research Project (SCCWRP).^[^ ^26^ ^]^ On the level of the International Organization for Standardization (ISO), standardization is ongoing for the international standards “Water quality – Analysis of microplastics in drinking water and groundwater–Part 2: Method using vibrational spectroscopy Vibrational spectroscopy methods for waters with low content of suspended solids including drinking water” (ISO/FDIS 16094‐2)^[^ ^27^ ^]^ and “Water quality–Analysis of microplastics in drinking water and groundwater–Part 3: Thermo‐analytical methods for waters with low content of suspended solids including drinking water” (ISO/DIS 16094‐3).^[^ ^28^ ^]^ Both proposals are nearly finished documents, which means on the level of (Final) Draft International Standard (DIS) and are expected to be finalized in 2025. Further projects related to sample preparation in general, extraction of MNPs from composts, and avoiding pellet loss are in progress, but on a lower processing level in standardization progress. This means that their completion will take another 2–3 years.

However, testing laboratories (including commercial labs and regulatory monitoring institutes) and academic research have different needs in relation to this particular analytical field. For testing laboratories, standards are a basic requirement for reliable measurements according to the client's needs, but research and practice showed that for the topic of “omnipresent MNPs” there is no “one size fits all” solution. For research, the aim should therefore be to harmonize research approaches as a preliminary step toward standardization. Harmonization of sampling, sample preparation, test materials, detection, and toxicological analysis should first be achieved and consolidated to make results comparable, which is also easier and quicker to achieve than standardization. The standardization process can be performed as a second step. Further on, research journals could also refer to harmonized protocols as a prerequisite for the acceptance of manuscripts.

Researchers from academia and industry should be heavily involved in the development of standardization, as both groups have the practical knowledge underpinning the methodology. In addition, industry can help in funding the development of standardized methods and of inter‐laboratory method evaluation studies (inter‐laboratory comparison, ILC).

Carrying out ILC is another hurdle in method validation. An ILC requires suitable reference materials, which should be homogeneous and stable as a minimum requirement. Furthermore, these should be available in sufficient quantities for different polymers, particle sizes, and shapes. So far, reference materials are far from being available. Although the number of research publications on MNPs is rapidly growing every year,^[^ ^29^, ^30^ ^]^ reference materials that are coordinated between labs for the most common polymers are still needed. For comparable research purposes, it is necessary that reference materials are reliable given the many different types of MNPs in terms of sizes, shapes, and surface characteristics. Apart from reference materials, harmonized analytical methods are needed. Existing analytical methods that have been commonly used in microplastics research (e.g., Raman spectroscopy, infrared spectroscopy, as well as pyrolysis‐gas chromatography–mass spectrometry, Thermal Extraction and—Desorption combined with Gas Chromatography‐Mass Spectrometry) can serve as a starting point to develop harmonized approaches to reliably detect and monitor MNPs.

A first ILC regarding MNPs analytics was carried out in 2020/2021 for the above‐mentioned standardized drinking water analysis in California. Twenty‐two laboratories from six countries participated in this study.^[^ ^26^ ^]^ At the same time, JRC (Joint Research Centre of the European Commission) in cooperation with BAM (Federal Institute for Materials Research and Testing, Germany) organized an international ILC on the quantification of microplastics in water with 94 participating laboratories.^[^ ^31^ ^]^ Further, in 2022 and 2023, BAM organized ILCs for the detection and characterization of a) microplastics and b) nanoplastics in the course of VAMAS (Versailles Project on Advanced Materials and Standards) activities funded by the EU Horizon 2020 project PlasticsFatE.^[^ ^32^ ^]^ Further ILCs are urgently needed to guide and support MNPs research and the development of reliable methods, but it is often difficult to secure appropriate funding for inter‐laboratory studies as they do not fit into typical “research projects”. In addition, this funding itself needs to be adapted to the long‐term timescales required to develop validated methods, as well as to provide the necessary infrastructures (e.g., through material repositories and databases). The data and knowledge gained will be transferred to future research. The open‐access database Toxicity of Microplastics Explorer (ToMEx) represents a first approach to exchange information and to keep an overview of the current research results.^[^ ^33^ ^]^

In order to do justice to their importance, MNPs should also be officially recognized as a new class of pollutants. Here, too, California can be cited as a first example, where consideration is being given to including microplastics in the candidate list of chemical pollutants, which would entail a strict evaluation of the corresponding products containing or releasing microplastics.^[^ ^34^ ^]^ However, for their monitoring, a number of hurdles must be overcome. Although first methods are proposed,^[^ ^26^ ^]^ routine analysis has been difficult to achieve until now. This is not only because a clean environment is required, especially when analyzing food or human tissues or blood, but also because monitoring (including sampling) methods for MNPs are still very challenging and often limited to specialized laboratories. Such laboratories often handle a large number of samples and are not easily accessible. We also need to decide which characteristics to monitor (such as polymer type, size, etc.) and if associated chemicals should also be analyzed. It also makes sense to select them according to the relevance of the risk potential of MNPs for humans or the environment. In the first instance, monitoring should focus on MNPs, which are most commonly produced, used, and widely spread in the human environment. Infrared and Raman spectroscopy can routinely and time‐effectively detect particles down to 10 and 1 µm, respectively.^[^ ^35^, ^36^ ^]^ In some cases, even smaller particles can be detected, for example, down to 0.05 µm by Raman tweezer.^[^ ^37^ ^]^ However, blind spots cannot be avoided, as they require specific equipment and skills, and the methods are only suitable for less complex matrices. Therefore, the detection of smaller particles (i.e., sub‐micron and nanoplastics), which are potentially ubiquitous in the environment, is often limited. In contrast to the particle counting methods, mass determining methods such as the chromatography–mass spectrometry related methods are not limited to the lower particle size. However, their limit is a critical amount of particle number with sufficient mass, which means in practice that a very high number of small particle is needed (each particle with a relatively small mass) or large particles (each particle with a relatively high mass). All described methods have in common the need for sophisticated equipment and well‐trained personnel. Both analytical approaches have different requirements for sample preparation. Using particle counting methods, a time‐intensive, nearly complete extraction of the matrix is needed to avoid the time‐consuming identification of non‐MNPs in the sample. Using chromatography–mass spectrometry related methods, the removal of the matrix can be obsolete or is less significant, however, risks of overplayed signals exist.

Nevertheless, it is important to initiate the monitoring of MNPs as soon as possible, e.g., by monitoring larger MP particles or selected abundant polymers, and await the development of less sophisticated or faster, novel methods.

### 3.2 Lessons Learnt From the Toxicology of MNPs

The characterization of the hazard potential of MNPs is impaired to a large extent by the often inadequate knowledge of the material composition of the materials. Reliable statements and conclusions on toxicological effects of MNPs are only possible if, for example, additives are known, i.e., specified by the manufacturers, and if a number of physico‐chemical parameters are available. In particular, additives can have a major impact on the results of toxicological tests, which is why we should also be aware of their effects on the human body and the environment. While the particles themselves may not pose significant risks, it is the additives within these particles that can have adverse effects on health. Understanding the impact of these additives is crucial for ensuring safety and implementing appropriate regulations.^[^ ^38^ ^]^ If the additives are not made transparent by the manufacturer, an alternative approach for hazard assessment would be to assume a mixture of the chemicals that are known to be used for the different categories of additives, like, e.g., stabilizers, antioxidants, or plasticizers by using databases like the collection of plastic monomers, additives, and processing aids compiled by Wiesinger et al.^[^ ^39^ ^]^ In addition, degradation products of MNPs and additives should be considered, as their toxicological profile may differ from the original components of the particles. But we must know in advance which degradation products may occur. For this reason, toxicology studies should also include end‐of‐life products and the fate of released plastic particles. In this context, there is a need for regulation to ensure the use of a life‐cycle approach that takes the construction phase, application, and end‐of‐life of polymers, additives, and their degradation products into account.

For further work to characterize the hazard potential of MNPs, priority should be given to toxicological tests on sentinel (bio‐indicator) species and to tests that focus on the effects on human health. [For examples of bio‐indicator species, see Ref. [40]] Tests should reflect worst‐case scenarios and sub‐chronic and long‐term effects. Due to the complexity and diversity of MNPs, it is likely that a large number of tests will be required to characterize the toxicological effects of different plastics in relation to the type of polymer, additives, particle sizes, and environmental fate.^[^ ^41^ ^]^ The large number of tests required also emphasizes the need for simple, cost‐effective, and sensitive tests to be developed for MNPs. To reduce the number and range of tests where animals are involved, animal‐free test methods should be developed when enough knowledge on the relevant test conditions has been gained. Moreover, as we have seen in the past, MNPs do not always produce effects in toxicological tests. This is why even such negative results should be made available because they can save time (and funding) in future investigations. Funding programs, for example, within the framework of EU projects, are therefore needed to support the publication of such results.

The publication of scientifically rather unspectacular results may also be helpful when it comes to the development of standardized procedures. Here, SOPs and test guidelines should be prioritized for publication.

However, as with MNPs analysis, harmonization of test methods is probably more suitable for research than standardization, and it will significantly improve the validity of studies. One idea to drive this forward could be to make the use of harmonized test methods mandatory for publications in scientific journals. As far as harmonized documentation is concerned, there is already a tailor‐made proposal for microplastics in the form of the “Reporting guidelines” developed by Cowger et al.^[^ ^42^ ^]^ These guidelines are intended to increase the reproducibility and comparability of MP studies and cover, among other aspects, sampling, sample preparation, microplastics identification and quantification, as well as parameters of toxicological tests. Such harmonized reporting guidelines are a first step on the way to harmonized methods for the above‐mentioned aspects of microplastics studies. If ways could be found to involve researchers from academia, service providers, and industry more closely in regulation and in the processes of harmonization and standardization, this would make the relevant work more attractive to the scientific community. In this way, the relevance of research results for regulation and governance could become more prevalent and visible in the scientific culture.

### 3.3 Findings Concerning Design and Labeling

Although a characterization of the risk associated with the use of plastics for human health and the environment is still incomplete, in particular due to unrealistic exposure conditions, which includes the need for realistic test materials, the discussions during the workshop revealed some basic precautionary measures that are recommended to reduce exposure and potential risks. For example, we could be guided by what we already know from the well‐documented safety profiles of many additives associated with plastics, such as plasticizers, stabilizers, and flame retardants. Their harmful effects can be influenced by factors such as chemical structure, concentration, and potential for leaching. Understanding these properties can help in selecting additives for plastics that minimize overall impact on human health and the environment.

Products should then only contain plastic materials with additives that have the least impact on human health or the environment. Simplification would be another principle that is already recognized in the context of recycling. This means that composite plastics should be avoided as their recycling is problematic. However, for many products and applications, a simplification strategy aiming for a single type of plastic or a single type of polymer is not suitable. On the other hand, for some products, it is worth considering not using plastic at all, e.g., in food packaging as provisioned in packaging‐free supermarkets, where product design without packaging or without plastic in the packaging component is already being achieved in a growing number of cases.^[^ ^43^ ^]^ [for an overview on systems of provision interventions see Ref. [44]]

Potentially critical events during production, use, and end‐of‐life identified by life cycle analyses (LCA) should receive more attention and trigger appropriate risk minimization measures. An already widely accepted strategy is to consider the end‐of‐life phase of a product as early as possible in the design phase—as well as facilitate mechanical or chemical recycling. A more holistic approach is now also called for in the “End plastic pollution”‐Resolution of the United Nations (UN), which should prepare for an international treaty.^[^ ^45^ ^]^ According to the resolution, the entire life cycle of plastics should be considered, and circular economy approaches should be established in the member states. In addition to suitable design criteria and a suitable waste management system, economic incentives are also possible, which have already proven their effectiveness in other cases. For example, experience with the deposit on cans (e.g., in Germany) has shown that the mass of products ending up in the environment can be reduced through political instruments and economic incentives. For plastic, this could also mean that by certain measures it gets a value that at least reflects the impact on the environment and health, as well as the associated social costs. In this way, less inflationary use of products and also a limitation of environmental damage can be achieved. Some studies have already looked at the social and environmental costs of plastics. These costs are still not included in the price of plastics, which makes it a highly profitable business.^[^ ^46^ ^]^ Merkl and Charles estimate that the global social cost of harm to health resulting from exposure to plastic‐related chemicals and gastrointestinal tract disorders caused by MNPs will exceed 100 billion US dollars per year.^[^ ^47^ ^]^ The social costs of environmental damage caused by plastic pollution are estimated at between 10 and 100 billion US dollars per year.^[^ ^47^ ^]^ However, the current estimates cover a wide range. In contrast to the figures from Merkel and Charles,^[^ ^47^ ^]^ Beaumont et al. put the annual loss in value of the benefits resulting from marine ecosystem services alone at between 500 and 2500 billion US dollars per year.^[^ ^48^ ^]^

With regard to mechanical recycling, it must be pointed out that this is not yet possible without some major disadvantages. In particular, in connection with the hazard potential of the materials, it should be mentioned that the number of recycling cycles is critical with regard to toxic additives, as these can accumulate in the material (e.g., heavy metals in polyvinyl chloride). Degradation products from previously used polymers and substances may also be contained in the recycled products. But a circular economy will not work without recycling. Therefore, different recycling strategies are needed for different polymers and waste stream types. Mechanical recycling, as provided for in the EU, should therefore only be used for plastics that do not contain critical additives and have only a negligible amount of potentially accumulated harmful substances. Further on, the efforts to collect or sort plastic materials by type must be stepped up.

In addition to the consequences for the design of plastics, labeling requirements were also discussed at the stakeholder workshop. First, it became clear that consumers have a right to know what is contained in plastics, especially with regard to some critical products such as baby bottles, where exposure of more sensitive groups is to be expected. And if the presence of certain substances in the plastics used cannot be accurately predicted, then the labeling should at least use weakened wording such as “may contain …” In any case, the labeling must be carefully designed so that it fulfils its task as providing neutral and at the same time comprehensible information about the ingredients and the possible risks of plastics in order not to frighten or confuse consumers.

As far as the European market is concerned, the industry is already preparing for comprehensive labeling as part of the European Commission's new Ecodesign for Sustainable Products Regulation (ESPR),^[^ ^49^ ^]^ which was proposed in 2022 and builds on the current Ecodesign Directive.^[^ ^50^ ^]^ The ESPR is intended to provide a framework for the definition of ecodesign requirements for almost all product categories. Labeling for plastics and products containing plastics could be implemented in conjunction with a product passport, which will become mandatory under the ESPR. The industry is currently developing a standard for a digital product passport. It should be transparent and contain LCA‐relevant information. Based on a database in which all products are listed, such a passport would contain information on intentionally added additives, for example.

However, it also became clear in the discussions that the industry needs more information and more clarity at the regulatory level for such new and comparatively comprehensive labeling. Moreover, it was pointed out that the collection of information for databases required for complex labeling could also increase the cost of plastics. In addition, a standard is needed for the labeling of plastics, which could be introduced by adapting the Regulation (EC) No 1272/2008 on the classification, labeling and packaging of substances and mixtures (CLP Regulation).^[^ ^51^ ^]^ This would provide the basis for the transparency needed to introduce a more comprehensive approach that takes into account the entire life cycle and all contingencies, as envisaged in the UN resolution to end plastic pollution.^[^ ^45^ ^]^

To meet consumers' need for information, the labeling should not only list the ingredients, but also provide information about the risks associated with exposure to plastics and associated chemicals known to be hazardous. How can a label provide information about these risks? A kind of traffic light visualization has been proposed, as implemented by “Nutri‐Score.”^[^ ^52^ ^]^ However, there are also concerns regarding practicability, as different types of plastics are used in a large number of products. In these cases, all ingredients of all plastics in the product must be taken into account for the score.

Finally, it is important to consider where the labeling information should be placed on the packaging. This is a particular problem for small products, specific products, and products where labeling could impair functionality. However, the labeling does not have to be placed directly on the product. It could be marketed together with the product on a separate label.

## 4 Conclusion

Plastics are integral to economic growth, yet we still struggle to understand their long‐term environmental and health impacts. Initiating discussions among researchers, stakeholders, plastic producers, and policymakers is essential. The organized workshop brought together key players to share insights and expertise on issues related to plastics.

From the discussion, it has become clear that the informative value of analytical methods is still hampered by a number of basic requirements. More extensive harmonization of the current approaches to particle analysis and toxicity testing could already help a great deal by making it possible to compare the results obtained. For reliable monitoring analysis, however, a higher normative level is required, so that standardization should be sought here. Standardization requires the support and activity of organizations such as ISO or the European Committee for Standardization (CEN), but also appropriate long‐term funding. For the development of standards, however, it is necessary for SOPs to be developed on a larger scale than before and for more ILCs to be carried out. This requires sufficient funding, and scientific journals should also facilitate the publication of protocols and comparative studies with suitable publication formats.

**Figure 1:**
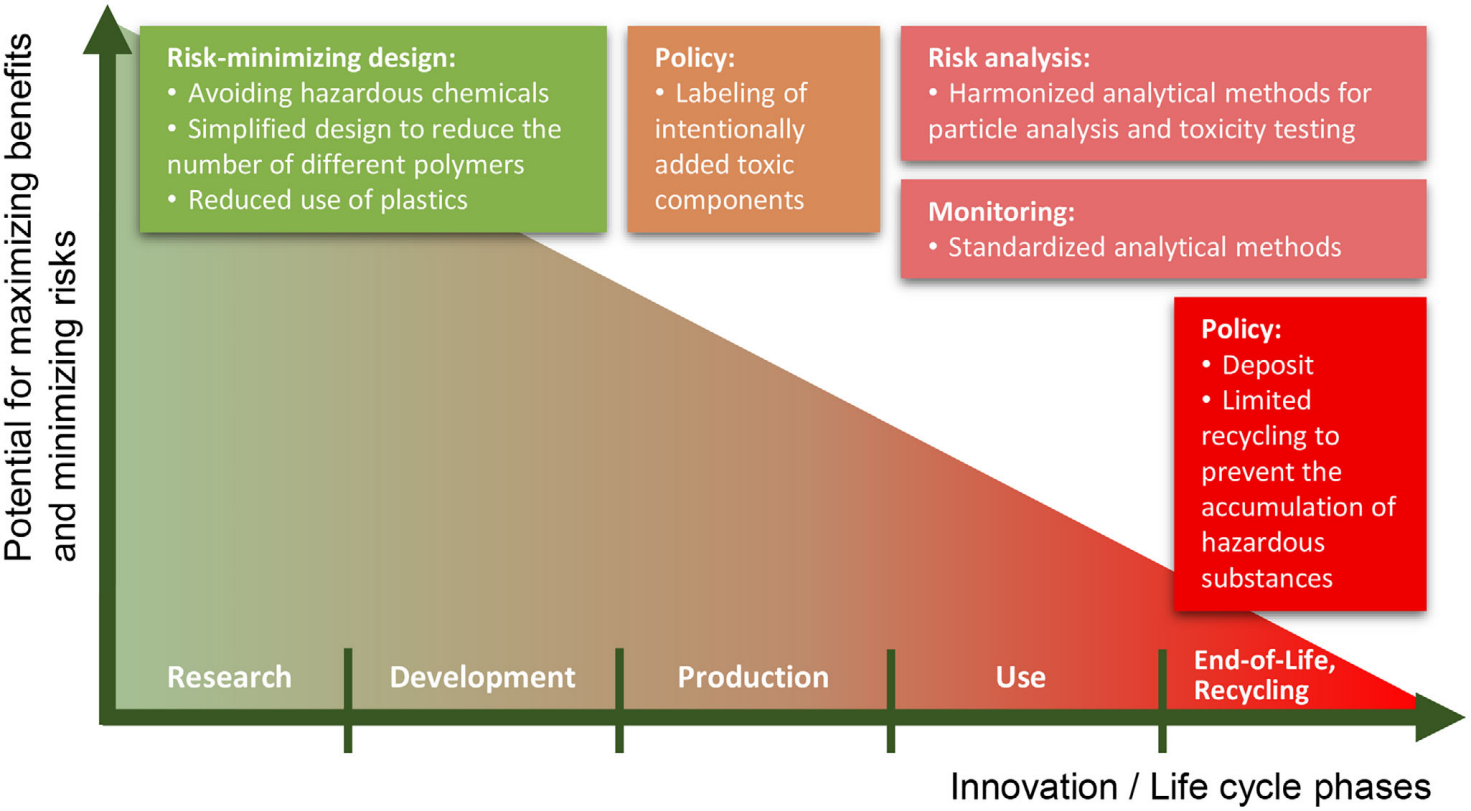
Key recommendations for risk reduction, analysis, and monitoring in relation to innovation phases and life cycle phases of plastics or plastic‐containing products.

The discussion on design and labeling offered possible solutions to avoid some of the current sources of risk associated with MNPs. In particular, it has become clear that a precautionary approach is needed that helps to avoid the mistakes made in the past. A risk‐minimizing design of plastics and products could be an early component of such a precautionary approach in the innovation process for new products (**Figure** **1**). This would mean, for example, that only substances with the least impact on human health and the environment would be considered. In addition, simplification through the elimination of plastics (e.g., in packaging) or the use of only one type of plastic would be very helpful. With regard to the end‐of‐life phase and recycling, policy instruments could consist of a deposit to increase the value of plastics and a restriction of recycling to plastics that do not accumulate pollutants and critical additives.

Labeling to inform users about critical aspects of the materials turned out to be a controversial topic, not least because our current understanding of possible human health implications is still rather limited. However, intentionally added toxic components of plastics should be made visible through appropriate labeling. In the EU, such labeling could potentially benefit from the efforts that are necessary anyway as part of the new EU Ecodesign Regulation.

If the precautionary principle,^[^ ^53^ ^]^ which is mandatory in EU technology policy for decades, is to apply to plastics, a regulatory approach is needed that covers all life cycle phases of plastics (e.g., production, distribution, use, and end‐of‐life) (Figure 1). Fortunately, the UN has already adopted such a far‐reaching and therefore responsible perspective in its resolution on plastic pollution,^[^ ^45^ ^]^ in which it also refers to the principles of the Rio Declaration on Environment and Development, which explicitly mentions the responsibility of states for sustainable production and consumption patterns and the prevention of damage to other states and territories.^[^ ^54^ ^]^ As with harmonization and standardization efforts, however, suitable funding programs are also needed here to prepare for the development of national and international regulations and their implementation by providing scientifically sound information.

## Conflict of Interest

The authors declare no conflict of interest.

## Data Availability

The corresponding author hereby declares that all data associated with the preparation of this manuscript are available from the authors.
